# Image-based robot assisted bicompartmental knee arthroplasty versus total knee arthroplasty

**DOI:** 10.1051/sicotj/2022048

**Published:** 2022-12-16

**Authors:** Jai Thilak, Srivatsa Nagaraja Rao, Vipin Mohan, Balu C. Babu

**Affiliations:** Department of Orthopaedics, Amrita Institute of Medical Sciences Kochi 682041 Kerala India

**Keywords:** Bicompartmental, Osteoarthritis, Robot-assisted, Arthroplasty

## Abstract

*Objective*: To evaluate the short-term clinical outcomes of image-based robot-assisted bicruciate retaining bicompartmental knee arthroplasty and compare it to robot-assisted total knee arthroplasty in the Indian population. *Methods*: Between December 2018 and November 2019, five patients (six knees) underwent robot-assisted bicompartmental knee arthroplasty (BCKA). These patients were demographically matched with five patients (six knees) who underwent robot-assisted total knee arthroplasty (TKA) during the same period. Clinical outcomes of these twelve knees were assessed in the form of knee society score (KSS) score, Oxford knee score (OKS), and forgotten joint score (FJS) after a minimum follow-up period of 25 months. The data between the two cohorts were compared and analyzed. *Results*: Scores obtained from both cohorts were subjected to statistical analysis. SPSS software was utilized and the Mann Whitney *U*-test was utilized to compare the two groups. There was no statistically significant difference found between the two groups in terms of functional outcome. *Conclusion*: Image-based robot-assisted BCKA is a bone stock preserving and more physiological procedure which can be a promising alternative to patients presenting with isolated arthritis of only two compartments of the knee. Although long-term, larger trials are warranted to establish it as an alternative, our pilot study shows an equally favorable outcome as TKA, making it an exciting new avenue in the field of arthroplasty.

## Introduction

The knee joint is one of the most common joints to be affected by osteoarthritis [1]. The final frontier of treatment for end-stage osteoarthritis of the knee is total knee arthroplasty (TKA) which is one of the most successful procedures with 82% survivorship reported at 25 years [2]. In total knee arthroplasty, all three compartments of the knee are resurfaced. However, all three compartments are not always affected in all cases of osteoarthritis. Medial compartment arthritis is 5–10 times more common than lateral compartment involvement [3]. A study done recently showed that of all patients awaiting total knee arthroplasty tricompartmental arthritis was found only in 16.7% whereas medial compartment arthritis was 51%, lateral compartment arthritis was 6.5% and patellofemoral arthritis was 1.2% [4]. A combination of medial and patellofemoral arthritis was found in 15.5% of patients [4]. In these patients, total knee arthroplasty not only sacrifices the problem-free compartment but also the anterior cruciate ligament which is pivotal in ensuring normal knee kinematics [5, 6]. Not to mention a greater loss of bone stock, making revision procedures difficult.

This has the led to development of a compartment-specific approach over the last decade which aims to resurface only the symptomatic and affected compartment(s). Unicondylar knee arthroplasty (UKA) is the most familiar procedure of the lot which has been reported to have outcomes similar to TKA [7]. Bicompartmental knee arthroplasty (BCKA) has similarly been practiced for resurfacing isolated medial and patellofemoral joint arthritis. Unlike TKA, it preserves both the cruciate ligaments and spares the unaffected compartment as well. It can be achieved by either using a single monolithic (monoblock) implant [8] or separate unlinked modular implants for the medial compartment and patellofemoral compartments. The unreliability of BCKA was primarily due to the studies on the older monolithic design implants which showed higher revision rates [9], limitation of flexion due to challenges in appropriate implant alignment, and reports of tibial plateau fractures [10]. There is no literature, to the best of our knowledge, on outcomes of robot-assisted BCKA using modular unlinked implants in the Indian population. The objective of this study is to determine any differences in short-term functional outcomes of patients undergoing robot-assisted BCKA versus robot-assisted TKA in an Indian population.

## Methods and materials

This was a pilot comparative cross-sectional study done at a quaternary care hospital in Kerala, India. Informed consent was obtained from all the participants. The first cohort consisted of five patients (*n* = 5, four female and one male) who had undergone robot-assisted bicompartmental knee arthroplasty between December 2018 and November 2019 with a mean age of 60.83 years (range 46–74 years) and a mean BMI of 28.87 (range 26–31.25). Of these patients, one underwent bilateral BCKA and the remaining four underwent unilateral BCKA. Inclusion criteria to undergo BCKA were patients who clinically had arthritic pain and examination findings confined to medial and patellofemoral compartments with no symptoms or signs in the lateral compartment and also had intact cruciate ligaments clinically. Long leg scanograms and dedicated knee radiographs were taken to confirm the presence of bicompartmental arthritic changes. All the patients had also undergone an MRI of the knee to confirm the integrity of cruciate ligaments, lateral meniscus and sparing of the lateral compartment.

These patients were matched demographically with five patients (*n* = 5, four females and one male) who underwent robot-assisted Cruciate-retaining total knee arthroplasty in the same period with a mean age of 61 years (range 53–71) and mean BMI of 29.2 (range 27–32.2). Of these patients, one underwent bilateral TKA and the remaining four underwent unilateral TKA. In both cohorts, patients who had a fixed flexion deformity of no more than 10°, a correctable varus deformity of up to 15°, with Grade III and IV osteoarthritis (Kellgren–Lawrence scale) were included. All the patients were ASA Grade-2 and had similar comorbidities.

**Figure 1:**
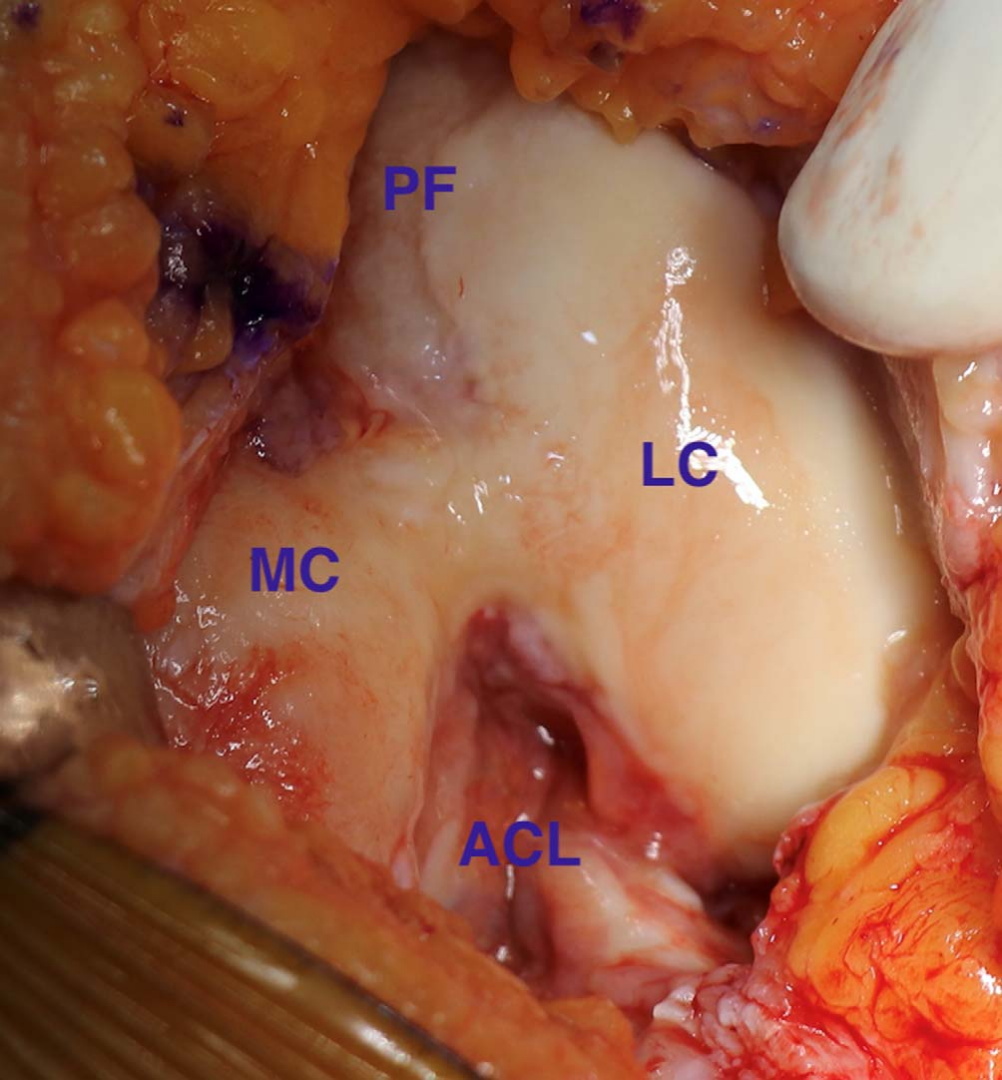
Intraoperative photograph showing medial compartmental and trochlear cartilage erosion in the knee with sparing of lateral compartment. PF = Patellofemoral compartment, LC = Lateral compartment, ACL = Anterior cruciate ligament, MC = Medial compartment.

**Figure 2:**
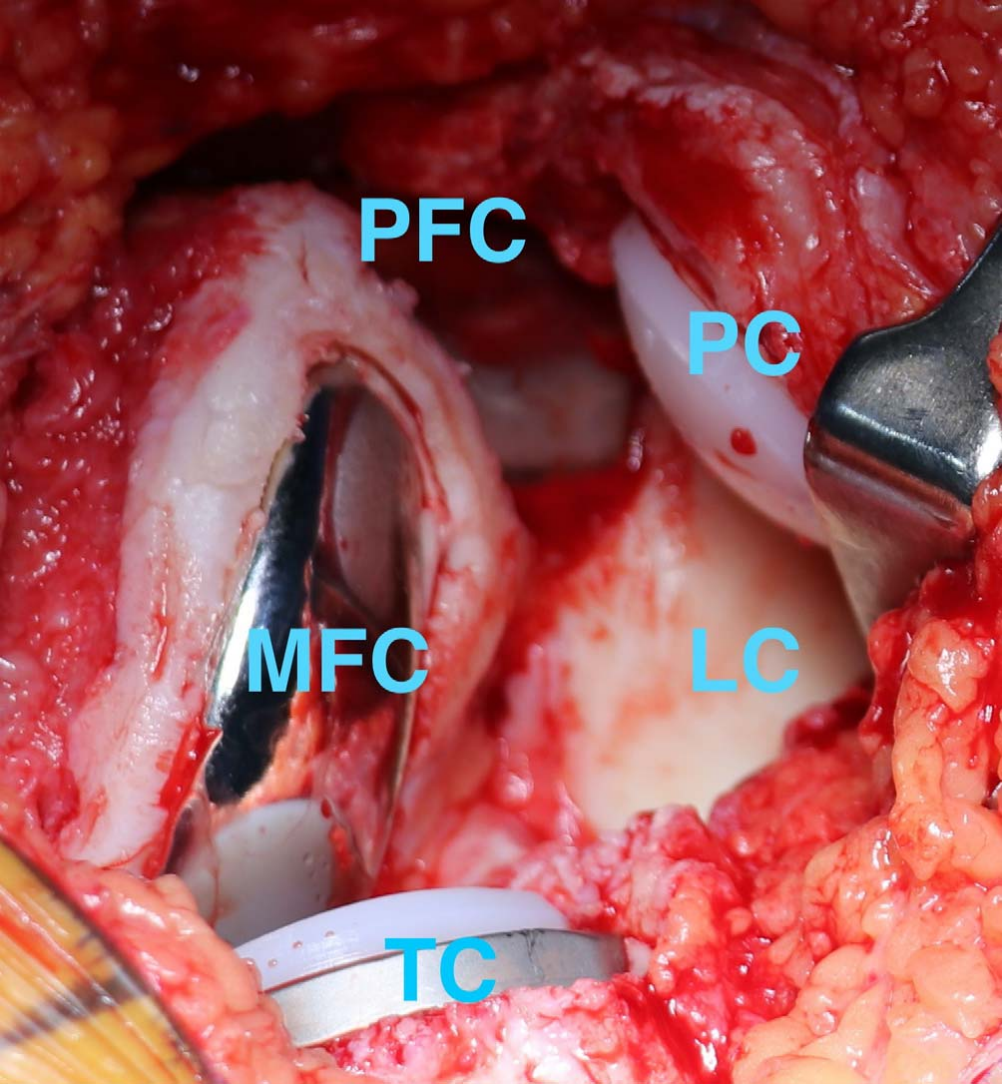
Intraop photograph showing final component position in a BCKA patient. PFC = Patellofemoral component, PC = Patellar component, LC = Lateral compartment, TC = Tibial component, MFC = Medial femoral component.

All the surgeries were by a single senior consultant surgeon and the robotic arm utilized was MAKO (Stryker^®^, USA) which is an image-based robotic system. A preoperative CT scan of the patient’s knee is done, segmented by a MAKO product specialist, and a 3D image is obtained. Based on this image, the surgeon is able to plan and optimize the component placement, and joint balance and minimize bony resection. One gram of intravenous tranexamic acid was administered prior to the inflation of the tourniquet. Standard anterior midline incision with a medial parapatellar approach was used for all surgeries. Intraoperatively once the exposure is done, real-time data on ligament balance is captured by the MAKO system, and readjustment of the surgical plan can still be done if needed. The robotic arm then aids the surgeon in resecting the planned amount of bone, followed by recapturing the ligament balance data after trial implantation. The implants utilized for BCKA were RESTORIS^®^ MCK bicompartmental implant system (Stryker^®^, USA) comprising of RESTORIS^®^ MCK femoral component, onlay tibial baseplate, onlay tibial insert, a patellofemoral component with Triathlon^®^ X3^®^ patella. The implants used for TKA were Triathlon^®^ total knee system (Stryker^®^, USA) with all patients having the patella replaced with Triathlon^®^ X3^®^ patella. BCKA was done as planned in the first cohort after intraoperative confirmation of intact lateral compartment cartilage and ACL (Figs. 1 and 2). No drain was placed postoperatively. Post-operative rehabilitation protocol was uniformly followed for both cohorts and patients were ambulated on the evening of surgery.

**Figure 3:**
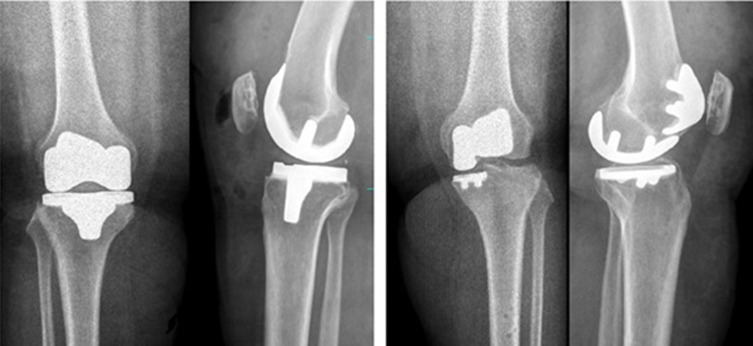
Post-operative knee X-rays of patients who underwent TKA (left-hand side) and BCKA (right-hand side).

All patients were followed up after a minimum period of 25 months with a mean follow-up period of 30.5 months (Fig. 3). Patient-reported outcome measures (PROMs) [11, 12] of knee society score – 2011 (KSS), Oxford knee score (OKS) and forgotten joint score (FJS) were obtained from both cohorts of patients and subject to statistical analysis using SPSS 17 software. The Mann-Whitney test was utilized to compare the continuous variables of the two cohorts. A *p*-value of less than 0.05 was considered statistically significant.

## Results

The mean follow-up period was 30.5 months postoperatively. The knee society score of both cohorts were analyzed under the components of symptoms, satisfaction score, expectation, and functional activity scores (Table 1). The BCKA group had a mean symptom score of 87.33 (SD = 10.25) with four patients having excellent outcomes and one patient having good outcomes (Table 1). The mean satisfaction score was 88 (SD = 0) with all five patients having the excellent outcome. The mean expectation score was 86.6 (SD = 0) with all five patients having the excellent outcome. The mean functional activity score was 71.3 (SD = 10.7) with one patient having an excellent outcome, three patients having a good outcome, and one patient having a poor outcome. The TKA group had a mean symptom score of 89.33 (SD = 10.58) with four patients having excellent outcomes and one patient having a good outcome. The mean satisfaction score was 87.5 (SD = 0) with all five patients having the excellent outcome. The mean expectation score was 86.6 (SD = 0) with all five patients having the excellent outcome. The mean functional activity score was 71.1 (SD = 12.5) with one patient having an excellent outcome, two having a good outcome, one having a fair outcome, and one having a poor outcome. There was no statistically significant difference between the BCKA and TKA cohorts in any of the KSS component scores (*p* > 0.05).

**Table 1:** Knee society scores of BCKA and TKA groups showing scores under different components. Scored out of 100. Grading: Score 80–100 Excellent; 70–79 Good; 60–69 Fair; Below 60 poor.

Knee society score (2011)									
BCKA group					TKA group				
Patient	Symptoms	Satisfaction	Expectation	Functional activity score	Patient	Symptoms	Satisfaction	Expectation	Functional activity score
A	80	88	87	70	A1	84	88	87	71
B	100	88	87	85	B1	100	88	87	85
C	100	88	87	75	C1	100	88	87	77
D	76	88	87	52	D1	76	88	87	52
E	84	88	87	73	E1	84	88	87	60
F	84	88	87	73	F1	84	88	87	60

Oxford knee scores were analyzed as functional (OKS-F) and pain (OKS-P) components between the two cohorts (Table 2). The OKS-F score in the BCKA group had a mean of 80 +/− 0 and the TKA group had a mean of 76.6 (SD = 5). There was no statistically significant difference between the OKS-F scores of the two groups (*p* = 1.06). The OKS-P score in the BCKA group had a mean of 96.98 (SD = 5.7) and the TKA group had a mean of 96.78 (SD = 5.1) (Table 2). There was no statistically significant difference between the OKS-F scores of the two groups (*p* = 1.34).

**Table 2:** Functional and pain component scores of OKS. Scored out of 100. Higher score indicates better outcomes and lower pain levels.

Oxford knee score							
Functional component				Pain component			
BCKA group		TKA group		BCKA group		TKA group	
Patient	Score	Patient	Score	Patient	Score	Patient	Score
A	80	A1	70	A	100	A1	100
B	80	B1	80	B	100	B1	100
C	80	C1	80	C	96	C1	100
D	80	D1	80	D	86	D1	86
E	80	E1	70	E	100	E1	93
F	80	F1	70	F	100	F1	93

Forgotten joint scores of the BCKA cohort had a mean of 67.7 (SD = 13.02) and the TKA cohort had a mean of 66.6 (SD = 10.67). There was no statistically significant difference between the FJS scores of the two groups (*p* = 2.8) (Table 3).

**Table 3:** Forgotten joint scores of both cohorts scored out of 100. Higher score indicates a better outcome.

Forgotten joint score			
BCKA group		TKA group	
Patient	Score	Patient	Score
A	75	A1	67
B	75	B1	75
C	69	C1	69
D	42	D1	42
E	75	E1	63
F	71	F1	63

None of the patients had any local or systemic perioperative complications such as cardiac/cerebrovascular events, urinary tract infections, and deep vein thrombosis. The mean operative time (skin incision to skin closure) in the BCKA group was 108.16 min versus 110.8 min in the TKA group. The surgeries were all carried out during the initial learning curve of robot-assisted surgery for the surgeon. All wounds healed well in the post-operative period and rehabilitation had been carried out as per standard protocol. Immediate post-op and follow-up radiographs showed implants in a good position with no evidence of any radiolucencies or implant loosening.

## Discussion

Compartment-specific approach to knee arthritis has evinced great interest in the field of arthroplasty and BCKA is increasingly being viewed as an alternative to TKA in a subset of patients. The standardized PROMs [11, 12] utilized showed marginally higher scores in the cohort of BCKA group but there was no statistically significant difference in the functional outcome between the two cohorts. BCKA is a bone stock and ligament-preserving surgery which is touted to be a significant plus point in better preserving knee kinematics, better gait, and patient satisfaction [9]. Concerns regarding the longevity of BCKA implants (58% at 17 years) were expressed by Parratte et al. [9] although this was based on the older implant designs utilized. The current generation of the modular unlinked prosthesis, especially with robotic arm assistance as utilized in our study, shows promising results in terms of implant design and component alignment which contributes greatly to survivorship, though long-term studies are awaited to precisely determine this. Image-based robotic technology such as the CT scan-based model derived from the MAKO^®^ system allows millimeter-level planning of component size and control of component placement with even one-degree accuracy [13]. Medial unicondylar knee arthroplasty (UKA) in patients with medial and patellofemoral arthritis features has shown to have poor outcomes as seen by Konan and Haddad [14] and these patients when critically selected can be the cohort that fits the bill for a BCKA. Yamabe et al. [15] demonstrated by using preoperative MRI scans that close to 41% of patients who underwent TKA could have been treated with BCKA or UKA. A recent study by Parratte et al. [16] showed that patients who underwent BCKA had greater FJS scores and better functional outcomes compared to those who underwent TKA at a two-year follow-up period. In addition, some studies have shown reduced intraoperative blood loss and thereby lower post-op blood transfusion rates in BCKA compared to TKA owing to less bone resection and soft tissue resection [17, 18]. Gait analysis on a treadmill by Garner et al. [19] has demonstrated an advantage of BCKA over TKA in top walking speeds and stride length as well owing to retention of the anterior cruciate ligament.

The results of our study are in agreement with existing literature which shows comparable outcomes between BCKA and TKA at a short-term follow-up. Tan et al. [20] assessed the two-year outcome of BCKA versus TKA and found that short-term results are comparable in terms of knee society scores, WOMAC, and SF-36 scores. Engh et al. [10] conducted a study that showed equivalent results in functional testing and clinical scores during a two-year follow-up period between BCKA and TKA. Blyth et al. [21] conducted a randomized trial between robotic arm-assisted BCKA and TKA and showed similar clinical outcomes at one-year post-op. Lower intraoperative blood loss and slightly better post-op range of motion in the BCKA group compared to TKA were found in a meta-analysis conducted by Elbardesy et al. [22]. Yeo et al. [17] went ahead to demonstrate equivalent clinical and functional scores at five years post-op with modular BCKA implants as compared to TKA. Schrednitzki et al. [23] showed equivalent scores between BCKA and TKA but with a better range of motion and less blood loss in the BCKA group. Biazzo et al. [24] conducted a match-paired study between BCKA and TKA groups and found that the clinical scores are equivalent and the results are comparable. They recommended BCKA as a viable alternative in mid-aged patients with bicompartmental arthritis as it is bone-stock preserving, thereby making a future revision easier if needed. A review of the comparative studies is provided in the table below:

**Table:** 

Author(s)	Journal/book	Conclusion
Blyth et al. [21]	Bone Joint J (2021)	Robot-assisted BCKA shows similar outcomes and post-op complications compared to TKA
Garner et al. [19]	KSSTA (2021)	BCKA results in nearer-normal gait and improved PROMs over TKA
Schrednitzki et al. [23]	J Arthroplasty (2020)	No significant differences in clinical scores, but better ROM in BCKA group
Biazzo et al. [24]	Musculoskeletal Surgery (2019)	BCKA theoretically better than TKA, can be offered as an alternative to TKA in young and high-demand patients
Kooner et al. [18]	Arthroplasty Today (2017)	Use of modular BKA for MPFOA is comparable with TKA in terms of short-term function, complication rate, and revision rate
Parratte et al. [16]	Orthop Traumatol Surg Res (2015)	After two years contemporary unlinked BKA was associated with greater comfort and better functional outcomes, compared to TKA
Engh et al. [10]	J Arthroplasty (2014)	Two years postoperatively the BKA and TKA groups achieved equivalent results in clinical scores and functional testing
Tan et al. [20]	J Orthop Surg (2013)	Short-term outcome of BCKA and TKA comparable

Bicompartmental knee arthroplasty is being increasingly seen as a viable and exciting avenue in the field of arthroplasty especially for younger patients with spared lateral knee compartments owing to its advantages. The available reports on short-term outcomes demonstrate equivalent outcomes with TKA, if not superior, thereby steering us logically to choose it for select patients who qualify for it. In addition the availability of robotic arm-assisted technology is encouraging for the accuracy it affords us in surgical planning and component placement [25, 26] which will go a long way in bettering clinical outcomes over the long term. The additional cost of preoperative CT scans, consumables, etc associated with robotic surgery can be justified as shown by Clement et al. [27] from health economic models. The current data on bicompartmental knee arthroplasty is outlined in the table below:

**Table:** 

Title	Author(s)	Journal/book	Conclusion
Bicompartmental knee arthroplasty [28]	Ries	Essentials of cemented knee arthroplasty (2022)	Favorable knee function and kinematics. Early failure may happen
The optimal indication for combined patellofemoral and unicondylar knee arthroplasty [29]	Beckmann and Meier	Basics in primary knee arthroplasty (2022)	Bicompartmental knee arthroplasty is a promising solution for young active patients with bicompartmental arthritis
Insufficient evidence to confirm benefits of custom partial knee arthroplasty: a systematic review [30]	Demey et al.	KSSTA (2021)	78–91% patient satisfaction after custom partial knee arthroplasty
Robotic-arm assisted bicompartmental knee arthroplasty: Durable results up to 7-year follow-up [31]	Gaudiani et al.	Int J Med Robot (2021)	Excellent survivorship, functional outcome, and good-excellent satisfaction at mid-term follow-up

The limitation of our study was the small number of patients, which possibly contributed to the lack of statistical significance. But this is a pilot study in the Indian subcontinent where robotic arm-assisted bicompartmental knee arthroplasty is only now coming of age. Longer term, large randomized clinical trials would give us a much more objective outlook on the feasibility of bicompartmental knee arthroplasty.
